# Inductive transfer learning for molecular activity prediction: *Next*-*Gen QSAR Models with MolPMoFiT*

**DOI:** 10.1186/s13321-020-00430-x

**Published:** 2020-04-22

**Authors:** Xinhao Li, Denis Fourches

**Affiliations:** Department of Chemistry, Bioinformatics Research Center, North Carolina State University, Raleigh, NC 27695 USA

**Keywords:** Transfer learning, Neural networks, Self-supervised learning, QSPR/QSAR

## Abstract

Deep neural networks can directly learn from chemical structures without extensive, user-driven selection of descriptors in order to predict molecular properties/activities with high reliability. But these approaches typically require large training sets to learn the endpoint-specific structural features and ensure reasonable prediction accuracy. Even though large datasets are becoming the new normal in drug discovery, especially when it comes to high-throughput screening or metabolomics datasets, one should also consider smaller datasets with challenging endpoints to model and forecast. Thus, it would be highly relevant to better utilize the tremendous compendium of unlabeled compounds from publicly-available datasets for improving the model performances for the user’s particular series of compounds. In this study, we propose the **Mol**ecular **P**rediction **Mo**del **Fi**ne-**T**uning (**MolPMoFiT**) approach, an effective transfer learning method based on self-supervised pre-training + task-specific fine-tuning for QSPR/QSAR modeling. A large-scale molecular structure prediction model is pre-trained using one million unlabeled molecules from ChEMBL in a self-supervised learning manner, and can then be fine-tuned on various QSPR/QSAR tasks for smaller chemical datasets with specific endpoints. Herein, the method is evaluated on four benchmark datasets (lipophilicity, FreeSolv, HIV, and blood–brain barrier penetration). The results showed the method can achieve strong performances for all four datasets compared to other *state*-*of*-*the*-*art* machine learning modeling techniques reported in the literature so far.
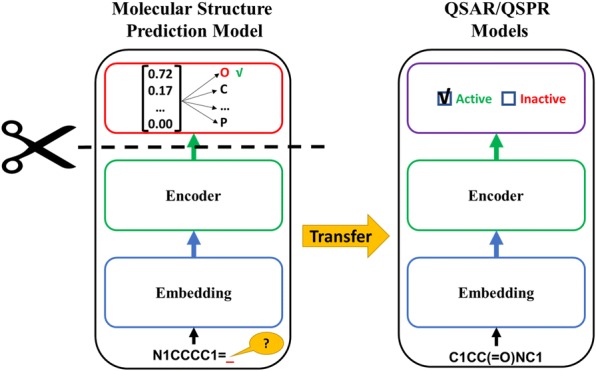

## Introduction

Predicting properties/activities of chemicals from their structures is one of the key objectives in cheminformatics and molecular modeling. Quantitative structure property/activity relationship (QSPR/QSAR) modeling [1–6] relies on machine learning techniques to establish quantified links between molecular structures and their experimental properties/activities. When using a classic machine learning approach, the training process is divided into two main steps: feature extraction/calculation and the actual modeling. The features (also called *descriptors*) characterizing the molecular structures are critical for the model performances. They typically encompass 2D molecular fingerprints, topological indices, or substructural fragments, as well as more complex 3D and 4D descriptors [7, 8] directly computed from the molecular structures [9].

Deep learning methods have demonstrated remarkable performances in several QSPR/QSAR case studies. In addition to use expert-engineered molecular descriptors as input, those techniques can also directly take molecular structures (*e.g.,* molecular graph [10–21], SMILES strings [22–24], and molecular 2D/3D grid image [25–30]) and learn the data-driven feature representations for predicting properties/activities. As a result, this type of approach is potentially able to capture and extract underlying, complex structural patterns and feature ↔ property relationships given sufficient amount of training data. The knowledge derived from these dataset-specific descriptors can then be used to better interpret and understand the structure–property relationships as well as to design new compounds. In a large scale benchmark study, Yang et al. [12] shown that a graph convolutional model that construct a learned representation from molecular graph consistently matches or outperforms models trained with expert-engineered molecular descriptors/fingerprints.

Graph convolutional neural networks (GCNN) directly operate on molecular graphs [10]. A molecular graph is an undirected graph whose nodes correspond to the atoms of the molecule and edges correspond to chemical bonds. GCNNs iteratively update the nodes representation by aggregating the representations of their neighboring nodes and/or edges. After *k* iterations of aggregation, the final nodes representations capture the local structure information within their *k*-hop graph neighborhood (which is somehow similar to augmented substructural fragments [31] but in a more data-driven manner). Moreover, the Simplified Molecular-Input Line-Entry System (SMILES) [32, 33] encodes the molecular structures as strings of text. Widely used in the field of cheminformatics, the SMILES format can be considered as an analogue of natural language. As a result, deep learning model architectures such as RNNs [34, 35], CNNs [36] and transformers [37] can be directly applied to SMILES for QSAR/QSPR tasks. While deep learning models have achieved *state*-*of*-*the*-*art* results on a variety of molecular properties/activities prediction tasks, these *end*-*to*-*end* models require very large amount of training data to learn useful feature representations. The learned representations are usually endpoint-specific, which means the models need to be built and retrained from scratch for the new endpoint/dataset of interest. Small chemical datasets with challenging endpoints to model are thus still disadvantaged with these techniques and unlikely to lead to models with reasonable prediction accuracy. As of today, this is considered as a grand challenge for QSAR modelers facing small sets of compounds without a clear path for obtaining reliable models for the endpoint of interest.

Meanwhile, transfer learning is a quickly emerging technique based on the general idea of reusing a pre-trained model built on a large dataset as the starting point for building a new, more optimized model for a target endpoint of interest. It is now widely used in the field of computer vision (CV) and natural language processing (NLP). In CV, a pre-trained deep learning model on ImageNet [38] can be used as the start point to fine-tune for a new task [39]. Transfer learning in NLP has historically been restricted to the *shallow* word embeddings: NLP models start with embedding layers initialized with pretrained weights from Word2Vec [40], GloVe [41] or fastText [42]. This approach only uses the *prior* knowledge for the first layer of a model, the remaining layers still need to be trained and optimized from scratch. Language model pre-training [43–47] extends this approach by transferring all the learned optimized weights from multiple layers, which providing *contextualized* word embeddings for the downstream tasks. Language scale pre-trained language models have greatly improved the performance on a variety of language tasks. The default task for a language model is to predict the next word given the past sequence. The input and labels of the dataset used to train a language model are provided by the text itself. This is known as *self*-*supervised learning*. Self-supervised learning opens up a huge opportunity for better utilizing unlabeled data.

Due to the limited amount and sparsity of labeled datasets for certain types of endpoints in chemistry (*e.g.,* inhibitor residence times, allosteric inhibition, renal clearance), several transfer learning methods have been developed for allowing the development of QSPR/QSAR models for those types of endpoints/datasets. Inspired by ImageNet pretraining, Goh et al. proposed ChemNet [26] for transferable chemical property prediction. A deep neural network was pre-trained in a supervised manner on the ChEMBL [48] database using computed molecular descriptors as labels, then fine-tuned on other QSPR/QSAR tasks. Jaeger et al. [49] developed Mol2vec which employed the same idea of Word2Vec in NLP. Mol2vec learns the vector representations of molecular substructures in an unsupervised learning manner. Vectors of closely related molecular substructures are close to each other in the vector space. Molecular representations are computed by summing up the vectors of the individual substructures and be used as input for QSPR/QSAR models. Hu et al. pre-trained graph neural networks (GNNs) using both unlabeled data and labeled data from related auxiliary supervised tasks. The pre-trained GNNs were shown to significantly increase the model performances [50]. Multitask learning (MLT) is a related field to transfer learning, aiming at improving the performance of multiple tasks by learning them jointly. Multitask DNNs (deep neural networks) for QSAR were notably introduced by the winning team in the Kaggle QSAR competition and then applied in other QSAR/QSPR studies [51–56]. MTL is particularly useful if the endpoints share a significant relationship. However, MTL requires the tasks to be trained from scratch every time.

Herein, we propose the **Mol**ecular **P**rediction **Mo**del **Fi**ne-**T**uning (**MolPMoFiT − **pronounced *MOLMOFIT*), an effective transfer learning method based on self-supervised pre-training + task-specific fine-tuning for QSPR/QSAR modeling. In the current version, a molecular structure prediction model (MSPM) is pre-trained using one million bioactive molecules from ChEMBL and then fine-tuned for various QSPR/QSAR tasks. This method is “*universal*” in the sense that the pre-trained molecular structure prediction model can be used as a source for any other QSPR/QSAR models dedicated to a specific endpoint and a smaller dataset (*e.g.,* molecular series of congeneric compounds). This approach could constitute a first look at next-gen QSAR models being capable of high prediction reliability even for small series of compounds and highly challenging endpoints.

## Methods

### ULMFiT

The MolPMoFiT method we proposed here is adapted from the ULMFiT (**U**niversal **L**anguage **M**odel **Fi**ne-**T**uning) [45], a transfer learning method developed for any NLP classification tasks. The original implementation of ULMFiT breaks the training process into three stages:

1. Train a general domain language model in the self-supervised manner on a large corpus (e.g., Wikitext-103 [57]). Language models are a type of model that aim to predict the next word in the sentences given the context precede it. The input and labels of the dataset used to train a language model are provided by the text itself. After training on millions of unlabeled text, the language model captures the extensive and in-depth knowledge [58–60] of a language and can provide useful features for other NLP tasks.
2. Fine-tuning the general language model on the task corpus to create a task specific language model.
3. Fine-tuning the task specific language model for downstream classification/regression model.

As described above, the ULMFiT is a three-stage transfer learning process that includes two types of models: language models and classification/regression models. A language model is a model that takes in a sequence of words and predicts the most likely next word. A language model is trained in a self-supervised manner and no label is required. This means the training data can be generated from a huge amount of unlabeled text data. The classification/regression model is a model that takes a whole sequence and predicts the class/value associated to the sequence, requiring labeled data.

### MolPMoFiT

In this study, we adapted the ULMFiT method to handle molecular property/activity prediction. Specifically, we trained a **molecular structure prediction model (MSPM)** using one million molecules extracted from ChEMBL with self-supervised learning. The pre-trained MSPM was then fine-tuned for the given QSAR/QSPR tasks.

**Fig. 1:**
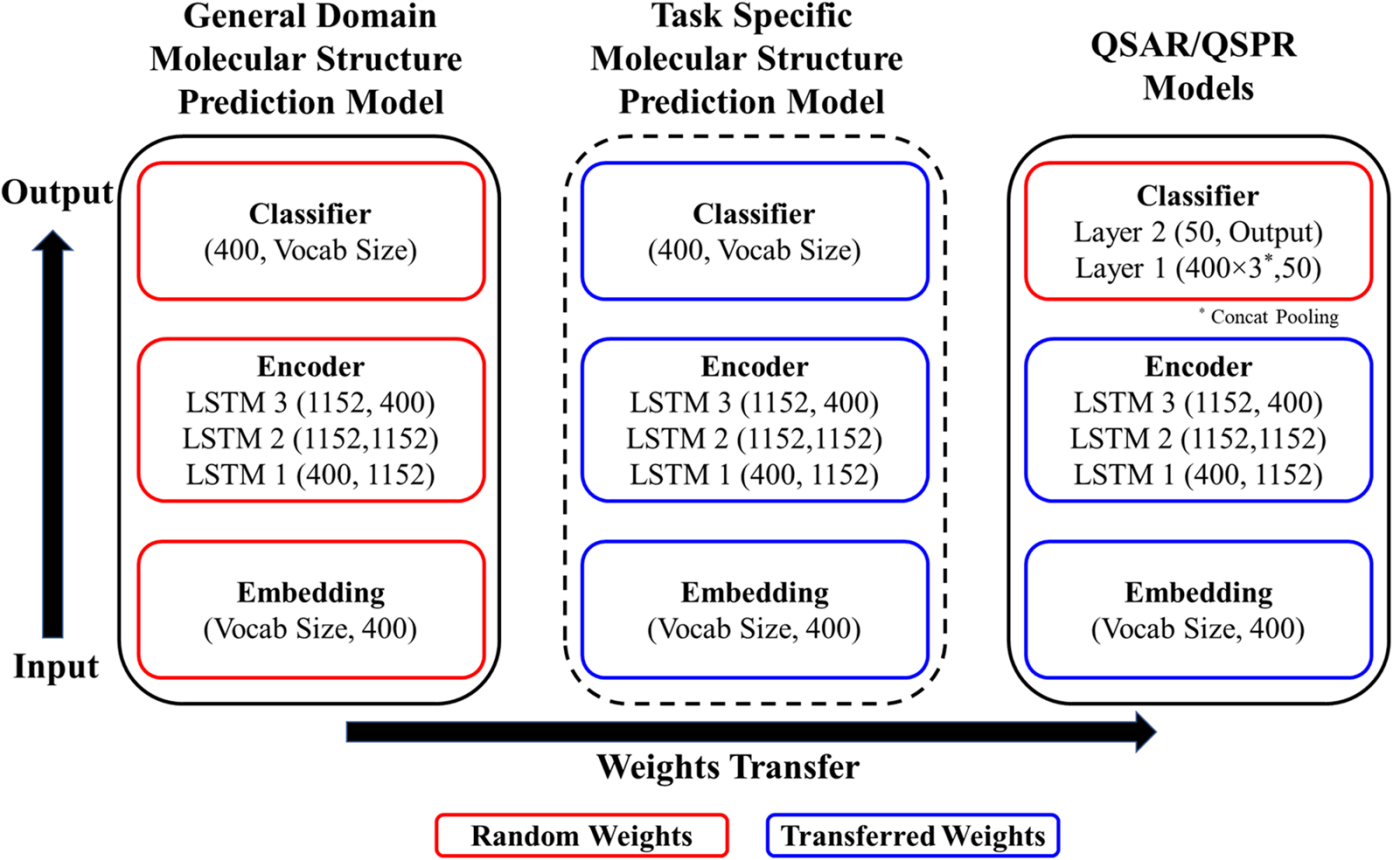
Scheme illustrating the **MolPMoFiT** Architecture: During the fine-tuning, learned weights are transferred between models. Vocab Size corresponds to the number of unique characters tokenized (see “Molecular representation” section) from SMILES in a data set. The stage of Task Specific Molecular Structure Prediction Model fine-tuning is optional

Model Architecture: The architectures for the MSPMs and the QSAR/QSPR models follow similar structures (Fig. 1): the embedding layer, the encoder and the classifier. The embedding layer converts the numericized tokens into fixed length vector representations (see “Molecular representation” section for details); the encoder processes the sequence of embedding vectors into feature representations which contain the *contextualized* token meanings; and the classifier uses the extracted feature representations to make the final prediction. The model architecture used for modeling is AWD-LSTM (ASGD Weight-Dropped LSTM) [61]. The main idea of the AWD-LSTM is to use a LSTM (Long Short-Term Memory [62]) model with dropouts in all the possible layers (embedding layer, input layer, weights, and hidden layers). The model hyperparameters are same as the ones initially implemented for ULMFiT. An embedding vector length of 400 was used for the models. The encoder consisted of three LSTM layers: the input size of first LSTM layer is 400, the hidden number of hidden units is 1152, and the output size of the last LSTM layer is 400. The classifiers use the output of the encoder to make predictions. The MSPMs and QSPR/QSAR models use the output of the encoder in different ways for different prediction purposes. The MSPM classifier consists of just a single softmax layer. The MSPMs predict the next token in a SMILES string, using the hidden state at the last time step *h*_*T*_ of the final LSTM layer of the encoder. The QSPR/QSAR model classifier consists of two feedforward neural network layers. The first layer takes the concatenation of output vectors from the last LSTM layer of the encoder (concatenation of max pooling, mean pooling and last time step *h*_*T*_ [45]), followed by a ReLU activation function. The final output size is determined by the QSPR/QSAR endpoints, *e.g.,* for regression models, a single output node is used; for classification models, the output size equals to the number of classes.

General-Domain MSPM Training: In the first stage of training, a general domain MSPM is trained on one million molecules curated from ChEMBL. The model is trained using the one cycle policy with a constant learning rate for 10 epochs. One cycle policy is a learning rate schedule method proposed by Smith [63]. The MSPM forms the source for all the subsequent QSPR/QSAR models. The training of the general-domain MSPM model requires about one day on a single NVIDIA Quadro P4000 GPU but it only needs to be trained once and can be reused for other QSPR/QSAR tasks.

Task Specific MSPM Model Fine-Tuning (Optional): The stage is optional for MolPMoFiT. The MSPM trained on ChEMBL covers a large and diverse chemical space of bioactive molecules and can be directly fine-tuned to predict physical properties of molecules such as lipophilicity and solubility. For bioactivities such as HIV inhibition and other drug activities, scientists are more interested in compounds with desired activities. The experimental tested data (target task dataset) may have a different distribution from ChEMBL. Fine-tuning the general domain MSPM on target task data to adapt to the idiosyncrasies of the task data would be helpful to the downstream QSAR models. The impact of task specific MSPM fine-tuning will be analyzed in “Benchmark” section.

In this stage, the goal is to fine-tuning the general domain MSPM on the target QSAR datasets to create the task-specific (endpoint-specific) MSPM. The initial weights (embedding, encoder and linear head) of task specific MSPM are transferred from the general domain MSPM. The task specific MSPMs are fine-tuned using the one cycle policy and discriminative fine-tuning [45]. In a neural network, different layers encode different levels of information [64]. Higher layers contain less general knowledge toward the target task and need more fine-tuning compared to lower layers. Instead of using the same learning rate for fine-tuning all the layers, the discriminative fine-tuning trains higher layers with higher learning rates. Learning rates are adjusted based on the same the function *η*^*layer* − *1*^ = *η*^*layer*^/2.6 used in the original ULMFiT approach, where *η* is the learning rate.

QSAR/QSPR Models Fine-Tuning: When fine-tuning the QSAR/QSPR model, only the embedding layer and the encoder are transferred from the pre-trained model, as the QSAR/QSPR model required a different classifier. In other word, the weights of classifier are initialized randomly and need to be trained from scratch for each task [45]. The QSPR/QSAR model is fine-tuned using one cycle policy, discriminative fine-tuning and gradual unfreezing [45]. During the fine-tuning, the model is gradually unfrozen over four layer-groups: (i) classifier; (ii) classifier + final LSTM layer; (iii) classifier + final two LSTM layers, and (iv) full model. Gradual unfreezing first trains the classifier of the model with the embedding and encoder layers frozen (weights are not updated). Then unfreezing the second to last layer-groups and fine-tuning the model. This process continues until all the layer-groups are unfrozen and fine-tuned.

Implementation. We implemented our model using the PyTorch [65] (https://pytorch.org/) deep learning framework and fastai v1 library [66] (https://docs.fast.ai). To ensure the reproducibility of this study, the data and code used in this study are freely available at: https://github.com/XinhaoLi74/MolPMoFiT.

### Dataset preparation

SMILES of all molecules in ChEMBL [48] were downloaded and curated following the procedure: (1) Removing mixtures, molecules with more than 50 heavy atoms (2) Standardizing with MolVS [67] package; (3) Sanitizing and canonizing with RDKit [68] package. After curation, one million SMILES were randomly selected for training and testing the molecular structure perdition model.

We tested our method on four publicly-available, benchmark datasets [15]: (1) molecular lipophilicity; (2) experimental measured solvation energy in kcal/mol (FreeSolv) (3) HIV inhibition, and (4) blood–brain barrier penetration (BBBP). The detailed descriptions are summarized in Table 1.

**Table 1:** Description of QSAR/QSPR datasets

Data Set	Description	Size	# of active compound	Task
*Lipophilicity*	Octanol/water distribution coefficient	4200		Regression
*FreeSolv*	Experimental measured solvation energy (kcal/mol)	642		Regression
*HIV*	Inhibition of HIV replication	41,127	1443	Classification
*BBBP*	Ability to penetrate the blood–brain barrier	2039	1560	Classification

### Molecular representation

In this study, we use SMILES strings as the textual representation of molecules. SMILES is a linear notation for representing molecular structures. For SMILES to be processed by machine learning models, they need to be transformed into numeric representations. SMILES strings are tokenized at the character level with a few specific treatments: (1) ‘Cl’, ‘Br’ are two-character tokens; (2) special characters encoded between brackets are considered as tokens (e.g., ‘[nH], ‘[O-]’ and ‘[Te]’ et al.). The unique tokens are mapped to integers to be used as input for the deep learning models.

### Data Augmentation

**Fig. 2:**
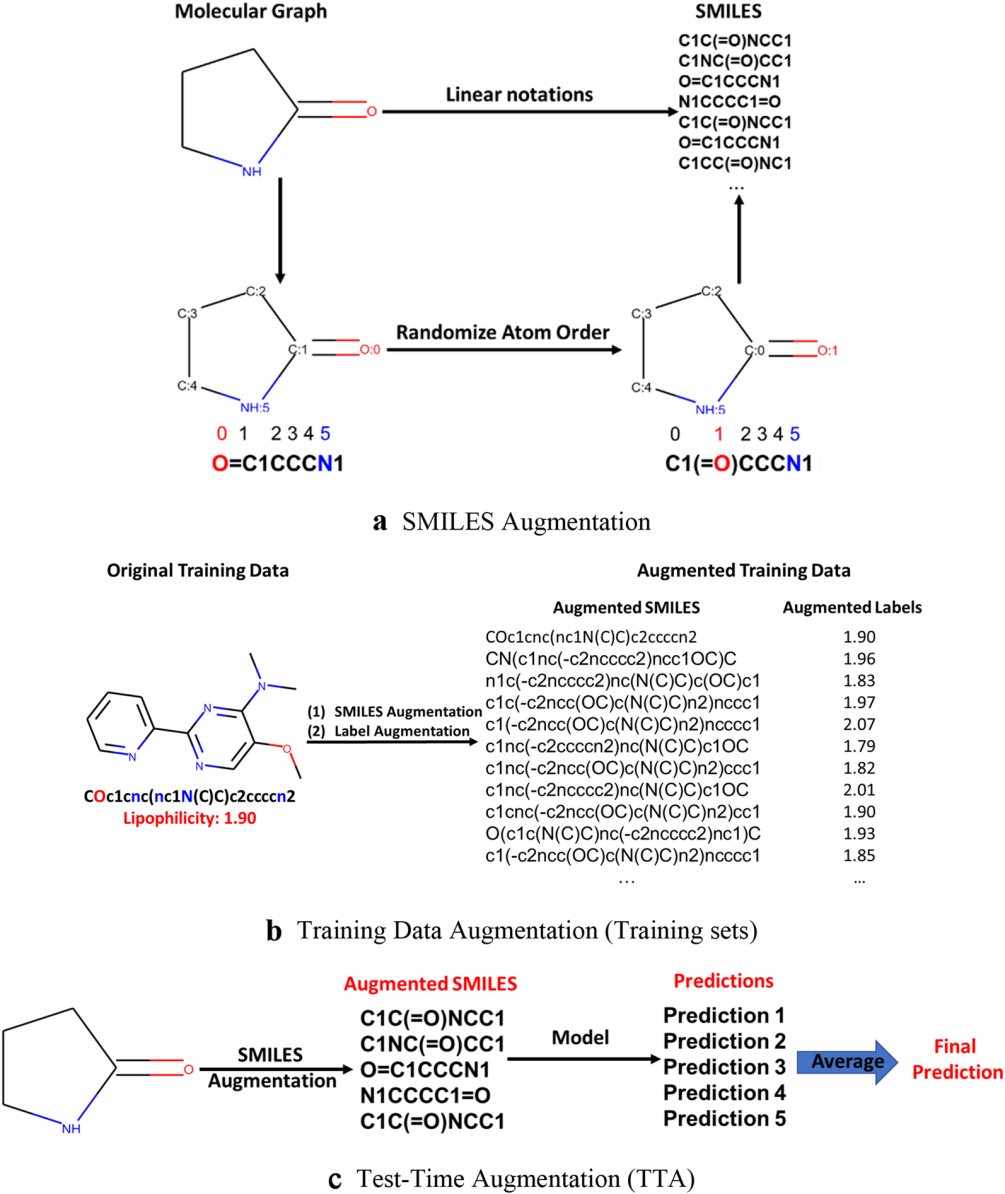
SMILES and Data Augmentation

Deep learning models are data-hungry so that various data augmentation techniques have been developed for different types of data and applications [69–72]. Data augmentation usually helps deep learning models to be better generalized for new data. Each SMILES corresponds to one unique molecular structure, whereas several SMILES strings can be derived from the same molecule. In fact, for a single molecular structure, many SMILES can be generated by simply randomizing the atom ordering (Fig. 2a). Bjerrum shown the SMILES enumeration as a data augmentation technique for QSAR models based on SMILES input can improve the robustness and accuracy [73]. It has been also shown that the generative models trained on both augmented and canonical SMILES can create a larger chemical space of structures [74, 75]. Herein, we used SMILES enumeration as the basis for data augmentation technique. The SMILES augmentation technique was applied to both the MSPM and QSAR/QSPR models. For MSPM, the SMILES augmentation ensures the trained model can cover a large and diverse chemical space (characterized by SMILES). For unbalanced classification QSAR/QSPR datasets, the SMILES augmentation can be applied to re-balance the class distribution of training data. In addition to SMILES augmentation, for regression QSAR/QSPR models, a Gaussian noise (mean set at 0 and standard deviation σ_noise_) is added to the labels of augmented SMILES which could be considered as a simulation of experimental errors [76]. (Figure 2b). The standard deviation σ_noise_ is considered as a hyperparameter for the models and need to be tuned from task to task. The impact of training data augmentation will be analyzed in “Analysis” section.

We also applied the test time augmentation (TTA): Briefly, the final predictions are generated by averaging predictions of the canonical SMILES and four augmented SMILES (Fig. 2c). The impact of TTA will be discussed in “Benchmark” section.

### Baselines and comparison models

To evaluate the performance of our method, we compared our models to the models reported by Yang et al. [12], including directed message passing neural network (D-MPNN), D-MPNN with RDKit features, random forest (RF) model on binary Morgan fingerprints, feed-forward network (FFN) on binary Morgan fingerprints, FFN on count-based Morgan fingerprints and FFN on RDKit descriptors,. We evaluated all models based on the original random and scaffold splits from Yang et al. for a fair and reproducible comparison. All the models were evaluated on the test sets on 10 randomly seeded 80:10:10 data splits. For regression model, we use root-mean-square-error (RMSE) as the key metric. For classification model, we use area under the receiver operating characteristic curve (AUROC) as the key metric.

### Hyperparameters and Training Procedure

QSAR/QSPR Model Fine-Tuning: We are interested in obtaining a model that perform robustly across a variety of QSPR/QSAR tasks. Herein, we used the same set of hyperparameters for fine-tuning QSPR/QSAR models across different tasks, which we tuned on the HIV dataset (Table 2). The batch size is set to 128 (64 for HIV dataset due to the GPU memory limit). The optimal hyperparameters of the HIV dataset was determined based on the validation set results on 3 randomly 80:10:10 data splits. Specifically, we optimized the dropout rates, the base learning rate and training epochs.

**Table 2:** Hyperparameters for QSPR/QSAR Model Fine-tuning

Layer groups	Base Learning Rate	Epochs
Linear head only	3e^−2^	4
Linear head + final LSTM layer	5e^−3^	4
Linear head + final two LSTM layers	5e^−4^	4
Full model	5e^−5^	6

Data Augmentation: In order to train a molecular structure prediction model that can be applied to a large chemical space, ChEMBL data is augmented by 4 times in addition to the original canonical SMILES. For the lipophilicity and FreeSolv datasets (regression), the number of augmented SMILES and the label noise σ_noise_ were tuned on the validation set on three 80:10:10 random split. Specifically, the SMILES of lipophilicity training data were augmented 25 times with the label noise σ_noise_ = 0.3 and the SMILES of FreeSolv training data were augmented 50 times with the label noise σ_noise_ = 0.5. For classification tasks, we used data augmentation to balance the class distribution. Specifically, for HIV data, the SMILES of active class were augmented 60 times and the SMILES of inactive class were augmented 2 times. For BBBP data, the SMILES of positive class were augmented 10 times and the SMILES of negative class were augmented 30 times.

## Results and discussion

### Benchmark

Yang et al. [12] developed a graph convolutional model based on directed message passing neural network (D-MPNN) and benchmarked it across a wide variety of public and proprietary datasets, achieved consistently strong performance. We benchmarked our MolPMoFiT method to the *state*-*of*-*the*-*art* models from Yang et al. on four well-studied chemical datasets: lipophilicity, FreeSolv, HIV and BBBP. Both random and scaffold splits were evaluated. Scaffold split enforced all training and test sets shared no common molecular scaffolds, which represent a more challenging and realistic evaluation compared to a random split. All the models were evaluated on test set on the exact same ten 80:10:10 splits from Yang et al. to ensure a fair and reproducible benchmark. Results for lipophilicity and FreeSolv data were evaluated by root mean square error (RMSE), whereas results for HIV and BBBP were evaluated by area under the receiver operating characteristic curve (AUROC). For physical properties lipophilicity and FreeSolv data, the regression models were fine-tuned on the general domain MSPM. For bioactivities HIV and BBBP data, the classification models were fine-tuned on both the general and task-specific MSPMs (see “MolPMoFiT” section). Evaluation metrics were computed in two settings: (1) testing on canonical SMILES only and (2) Time-time augmentation (TTA, see “Data augmentation” section).

**Fig. 3:**
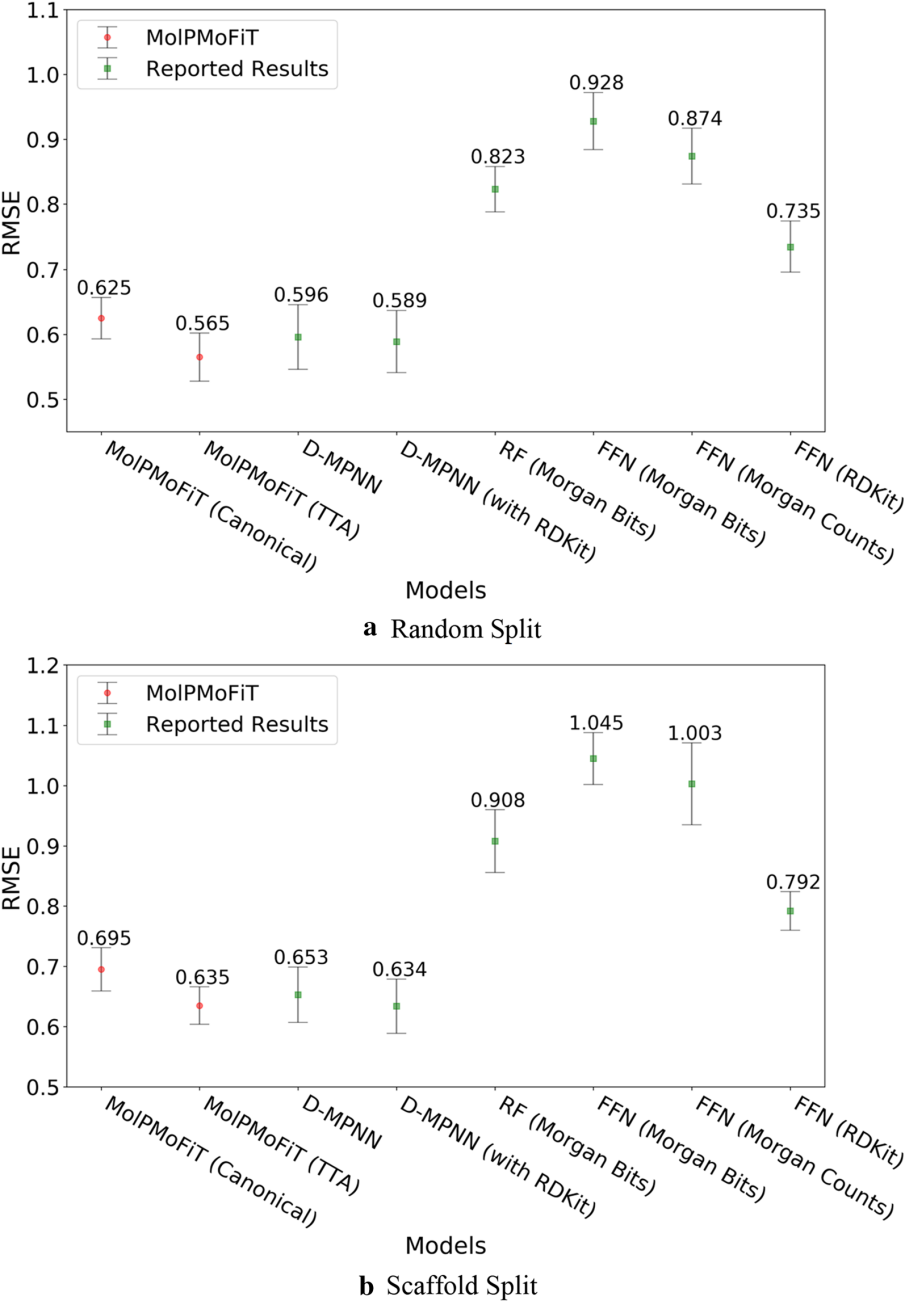
Comparison of MolPMoFiT to Reported Results from Yang’s [12] on Lipophilicity. **a** Random split; **b** Scaffold split. *MolPMoFiT* Molecular Prediction Model Fine-Tuning, *D-MPNN* Directed Message Passing Neural Network, *RF* Random Forest, *FFN* Feed-Forward Network

**Fig. 4:**
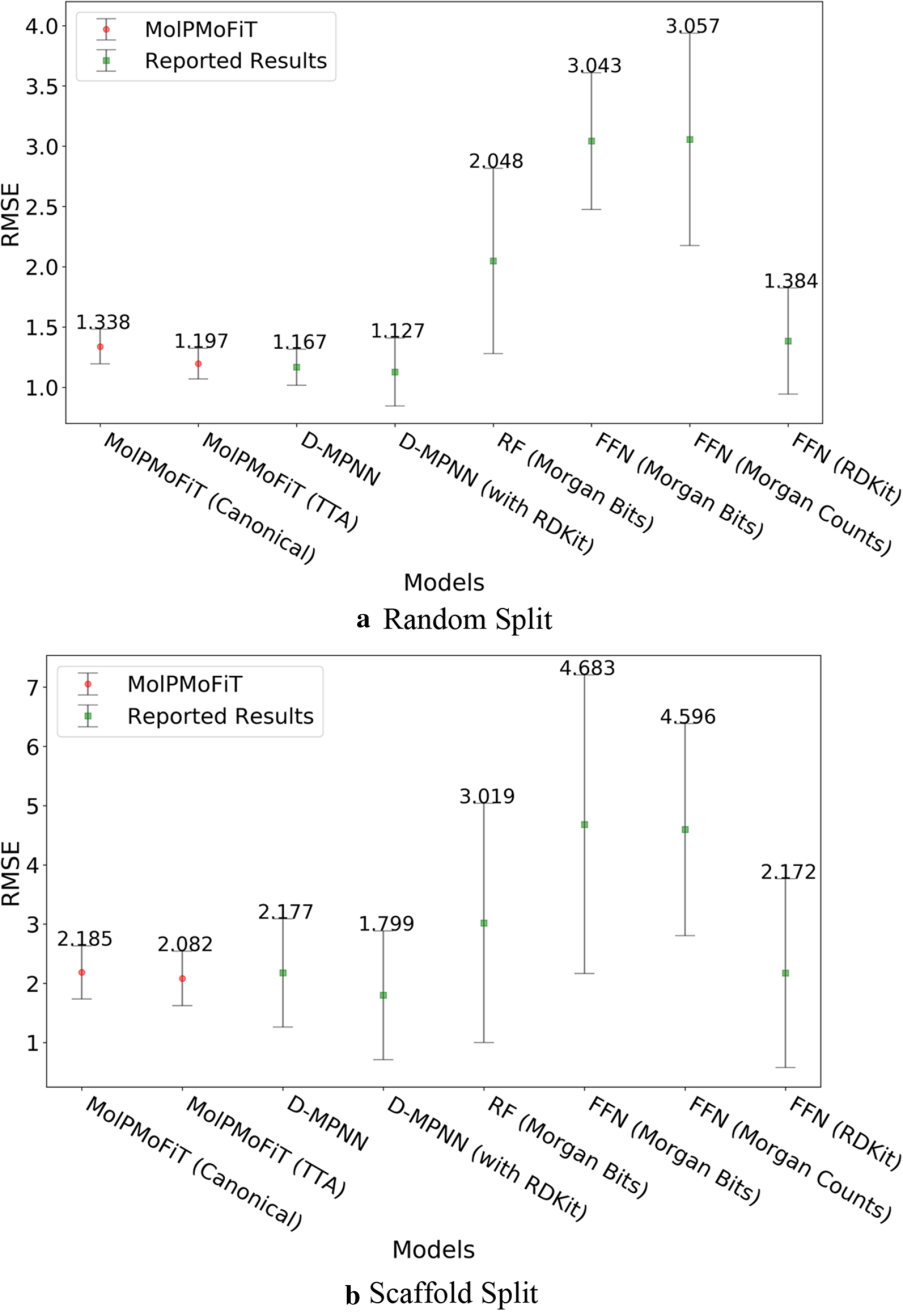
Comparison of MolPMoFiT to Reported Results from Yang’s [12] on FreeSolv. **a** Random split; **b** Scaffold split

**Fig. 5:**
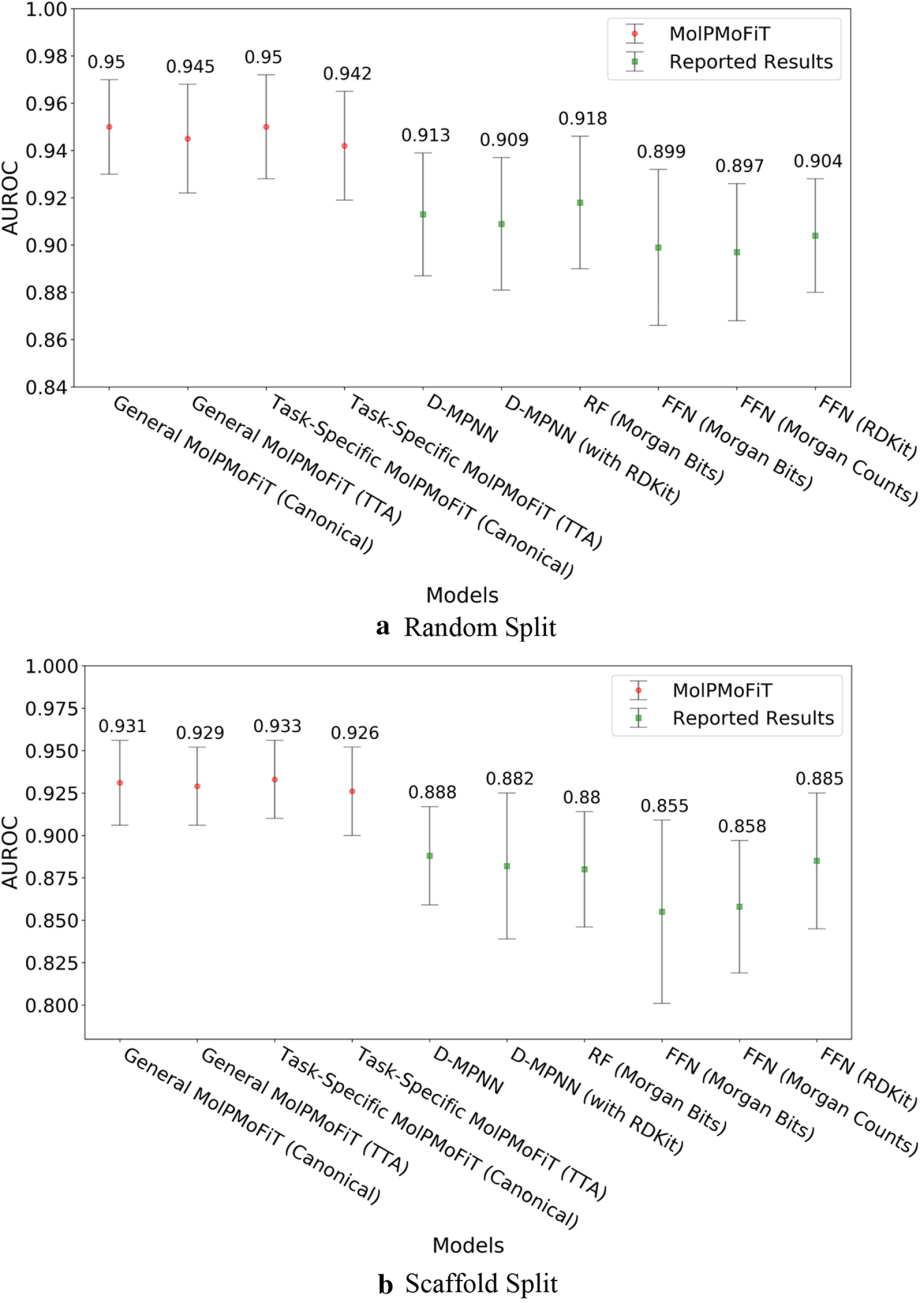
Comparison of MolPMoFiT to Reported Results from Yang’s [12] on BBBP. **a** Random split; **b** Scaffold split

**Fig. 6:**
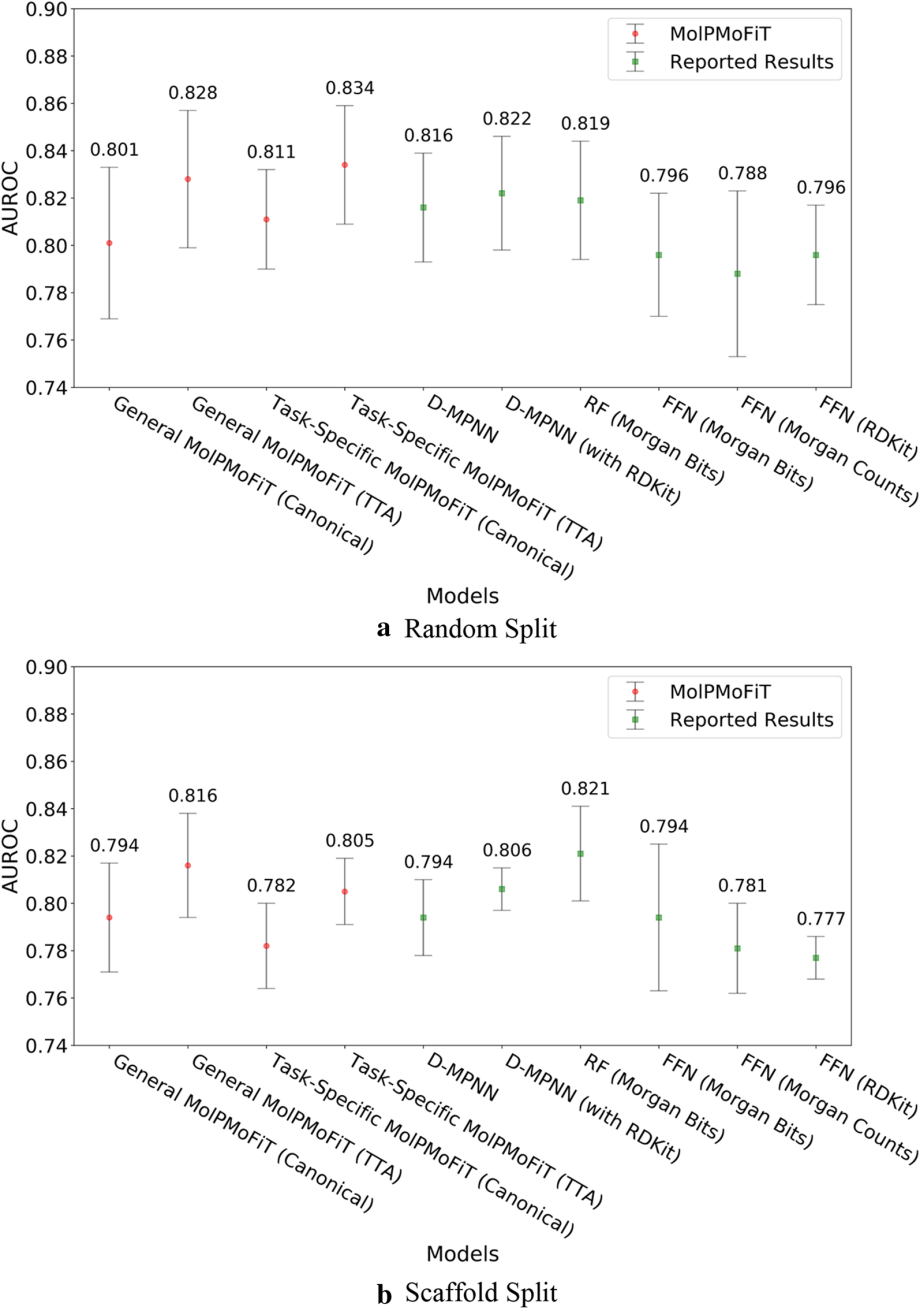
Comparison of MolPMoFiT to Reported Results from Yang’s [12] on HIV. **a** Random split; **b** Scaffold split

The results for test sets are summarized in Figs. 3, 4, 5 and 6. Across all four data sets, MolPMoFiT models achieved comparable or better prediction performances compared to the baselines. Generally, a scaffold split resulted in a worse performance compared to a random split. But a scaffold split can better measure the generalization ability of a model, which is very useful [77] for new molecular series with scaffolds being dissimilar to any other compounds in the modeling set.

For lipophilicity data, MolPMoFiT models tested on TTA outperform those tested on canonical SMILES. On random split, MolPMoFiT achieved a test set RMSE of 0.565 ± 0.037 and 0.625 ± 0.032 with and without TTA, respectively (Fig. 3a). On scaffold split, MolPMoFiT achieved a test set RMSE of 0.635 ± 0.031 and 0.695 ± 0.036 with and without TTA, respectively (Fig. 3b).

The FreeSolv dataset only contains 642 compounds, different data splits resulted in a large variance in RMSE (Fig. 4). MolPMoFiT models tested on TTA outperform those tested on canonical SMILES on random split but have no significant difference on scaffold split. On random split, MolPMoFiT achieved a test set RMSE of 1.197 ± 0.127 and 1.338 ± 0.144 with and without TTA, respectively (Fig. 4a). On scaffold split, MolPMoFiT achieved a test set RMSE of 2.082 ± 0.460 and 2.185 ± 0.448 with and without TTA, respectively (Fig. 4b).

For bioactivities like BBBP and HIV inhibition, the molecules of interest (tested experimentally) may have a different distribution from ChEMBL. Fine-tuning the general domain MSPM on target task data to adapt to the idiosyncrasies of the task data would be helpful to the downstream QSAR models. We evaluated the QSAR models fine-tuned both on general domain MSPM (named as general MolPMoFiT) and task-specific MSPM (named as task-specific MolPMoFiT) on BBBP and HIV datasets. For both BBBP (Fig. 5) and HIV (Fig. 6) dataset, the performance of models fine-tuned on general domain MSPM is on-par with the performance of models fine-tuned on the task-specific MSPMs. It requires more case studies to show whether fine-tuning on task-specific MSPM is beneficial.

On BBBP dataset, MolPMoFiT models outperform other comparison models (Fig. 5). Specifically, the general MolPMoFiT models achieved a test set AUROC of 0.950 ± 0.020 (Canonical SMILES) and 0.945 ± 0.023 (TTA) on random split and achieved a test set AUROC of 0.931 ± 0.025 (Canonical SMILES) and 0.929 ± 0.023 (TTA) on scaffold split. The task-specific MolPMoFiT models achieved a test set AUROC of 0.950 ± 0.022 (Canonical SMILES) and 0.942 ± 0.023 (TTA) on random split and achieved a test set AUROC of 0.933 ± 0.023 (Canonical SMILES) and 0.926 ± 0.026 (TTA) on scaffold split. It is worth noting that the implementation of TTA shows no improvements of the model accuracy.

For HIV data (Fig. 6), the general MolPMoFiT models achieved a test set AUROC of 0.801 ± 0.032 (Canonical SMILES) and 0.828 ± 0.029 (TTA) on random split and achieved a test set AUROC of 0.794 ± 0.023 (Canonical SMILES) and 0.816 ± 0.022 (TTA) on scaffold split. The task-specific MolPMoFiT models achieved a test set AUROC of 0.811 ± 0.021 (Canonical SMILES) and 0.834 ± 0.025 (TTA) on random split and achieved a test set AUROC of 0.782 ± 0.018 (Canonical SMILES) and 0.805 ± 0.014 (TTA) on scaffold split.

### Analysis

**Fig. 7:**
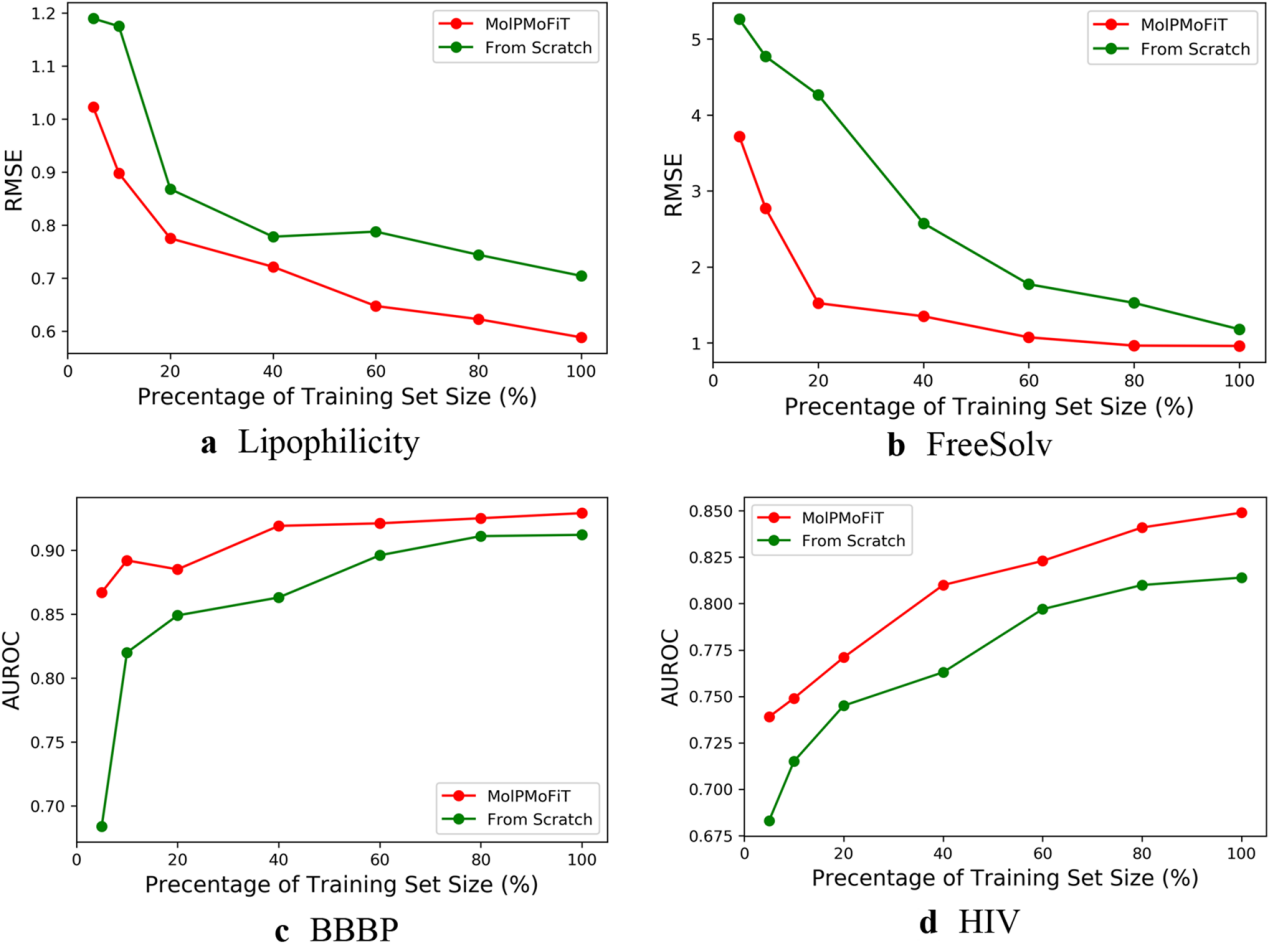
Performances of models on the different size of the training set. **a** Lipophilicity; **b** FreeSolv; **c** BBBP and **d** HIV

Impact of Transfer Learning: MolPMoFiT models were compared to the models that were trained from scratch. The models were trained on different number of training data and tested on the test set on a single 80:10:10 random split. The hyperparameters (learning rate, dropout rate and training epochs) were kept fixed: the hyperparameters of MolPMoFiT models were the same as we used in benchmark and the hyperparameters of models trained from scratch were tuned based on the validation set using the full training set. The results are illustrated in Fig. 7. Generally, with different numbers of training data, the MolPMoFiT model always outperforms the model trained from scratch. This indicated that the MolPMoFiT transfer learning technique provided a robust improvement for the model performances.

Impact of Training Data Augmentation: In “Benchmark” section, we shown that test-time augmentation (TTA) can improve the accuracy of predictions. Herein, we analyze the effect of training data augmentation. All models were evaluated with evaluation metrics computed with TTA on the test sets on three 80:10:10 random splits. The hyperparameters of models were the same as we used in benchmark.

For classification tasks (BBBP and HIV), models were trained on different sizes of augmented training data. On HIV dataset, when model trained on the original dataset (no augmentation), the AUROC is 0.816 ± 0.005, which is significantly low than those with data augmentation. The models achieved similar performance when data augmentation applied no matter class re-balancing or not (Table 3). Similarly, training data augmentation significantly improves the accuracy of the model on BBBP data. The model shows no improvement with the class re-balancing (Table 4).

**Table 3:** Impact of SMILES Augmentation on HIV Dataset

Iteration of augmentation		Class ratio (positive: negative)	AUROC
Positive class	Negative class		
0	0	0.037	0.816 ± 0.005
4	4	0.037	0.831 ± 0.003
30	1	0.52	0.830 ± 0.007
60	1	1	0.835 ± 0.007

**Table 4:** Impact of SMILES augmentation on BBBP dataset

Iteration of augmentation		Class ratio (positive: negative)	AUROC
Positive class	Negative class		
0	0	3.38	0.894 ± 0.004
4	4	3.10	0.937 ± 0.005
10	10	3.25	0.949 ± 0.002
9	30	1.1	0.946 ± 0.002

**Fig. 8:**
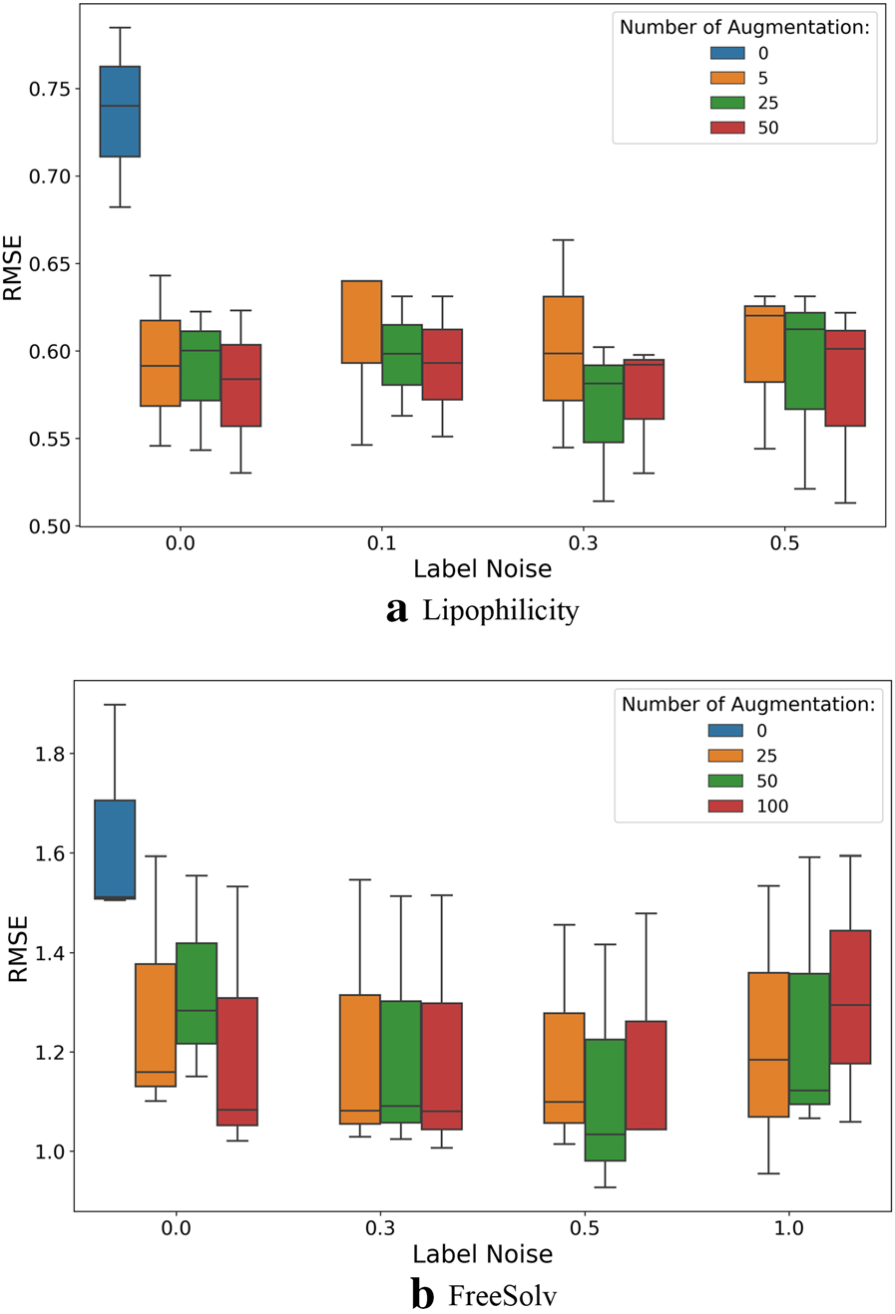
Performances of Lipophilicity models on different number of augmented SMILES per compound and Gaussian Noise (σ_noise_) added to the original experimental values

For regression tasks (lipophilicity and FreeSolv), models were trained on different sizes of augmented training data, whose labels were perturbed with different Gaussian noise σ_noise_. The evaluated numbers of augmented SMILES per compound were {0, 5, 25, 50} and {0, 25, 50, 100} for lipophilicity and FreeSolv, respectively. The evaluated Gaussian noise σ_noise_ values were {0, 0.1, 0.3, 0.5}and {0, 0.3, 0.5, 1} for lipophilicity and FreeSolv, respectively. The results on the test set are shown in Fig. 8. For both lipophilicity and FreeSolv datasets, when the model was only trained on the original training data (no augmented SMILES and perturbed labels), the performance is significantly worse than those of the models trained on augmented training data.

The results above show one limitation of using SMILES as input for deep learning model: the model actually learns to map individual SMILES to molecular properties/activities instead of linking actual molecular structures to their properties/activities. However, the SMILES augmentation is used as a regularization technique, making the model more robust to various SMILES representation for the same molecule. Appropriately adding random label noise to the augmented SMILES led to improved predictive power of the regression model. For the same data augmentation setting, testing results with TTA were found to be almost always better than the results on only canonical SMILES. While augmentation for training set can help in building models that can generalize well on new data, prediction accuracy can be further improved by TTA.

## Conclusions

In this study, we introduced the MolPMoFiT, a novel transfer learning method for QSPR/QSAR tasks. We pre-trained a molecular structure prediction model (MSPM) using one million bioactive molecules from ChEMBL and then fine-tuned it for various QSPR/QSAR tasks. This pre-training + fine-tuning approach enables knowledge learned from large chemical data sets to transfer to smaller data sets, thereby improving the model performance and generalization. Without endpoint-specific hyperparameter tuning, this method showed comparable or better results compared to that of the *state*-*of*-*the*-*art* results reported in the literature for four benchmark datasets. In addition to the strong *out*-*of*-*box* performance, this method reuses the pre-trained MSMP across QSPR/QSAR tasks so that reduces the burden of hyperparameters tuning and model training. We posit that transfer learning techniques such as MolPMoFiT could significantly contribute in boosting the reliability of next-generation QSPR/QSAR models, especially for small/medium size datasets that are extremely challenging for QSAR modeling.

## Data Availability

The curated datasets (.smi and .csv files) and the full updated code used in this study are freely-available at: https://github.com/XinhaoLi74/MolPMoFiT.

## Abbreviations

MolPMoFiT: **Mol**ecular **P**rediction **Mo**del **Fi**ne-**T**uning
QSPR/QSAR: Quantitative structure property/activity relationship
SMILES: Simplified Molecular-Input Line-Entry System
GCNN: Graph convolutional neural networks
RNN: Recurrent neural network
CNN: Convolutional neural network
CV: Computer vision
NLP: Natural language processing
MLT: Multitask learning
MSPM: Molecular structure prediction model
ULMFiT: **U**niversal **L**anguage **M**odel **Fi**ne-**T**uning
AWD-LSTM: ASGD Weight-Dropped LSTM
D-MPNN: Directed Message Passing Neural Network
RF: Random Forest
FFN: Feed-Forward Network
TTA: Test-time augmentation
